# Inflammatory checkpoints in amyotrophic lateral sclerosis: From biomarkers to therapeutic targets

**DOI:** 10.3389/fimmu.2022.1059994

**Published:** 2022-12-22

**Authors:** Zongzhi Jiang, Ziyi Wang, Xiaojing Wei, Xue-Fan Yu

**Affiliations:** Department of Neurology and Neuroscience Center, The First Hospital of Jilin University, Changchun, China

**Keywords:** amyotrophic lateral sclerosis, animal model, biomarker, inflammation, immunotherapy

## Abstract

Amyotrophic lateral sclerosis (ALS) is a neurodegenerative disease characterized by progressive motor neuron damage. Due to the complexity of the ALS, so far the etiology and underlying pathogenesis of sporadic ALS are not completely understood. Recently, many studies have emphasized the role of inflammatory networks, which are comprised of various inflammatory molecules and proteins in the pathogenesis of ALS. Inflammatory molecules and proteins may be used as independent predictors of patient survival and might be used in patient stratification and in evaluating the therapeutic response in clinical trials. This review article describes the latest advances in various inflammatory markers in ALS and its animal models. In particular, this review discusses the role of inflammatory molecule markers in the pathogenesis of the disease and their relationship with clinical parameters. We also highlight the advantages and disadvantages of applying inflammatory markers in clinical manifestations, animal studies, and drug clinical trials. Further, we summarize the potential application of some inflammatory biomarkers as new therapeutic targets and therapeutic strategies, which would perhaps expand the therapeutic interventions for ALS.

## Introduction

Amyotrophic lateral sclerosis (ALS) is a heterogeneous neurodegenerative disease characterized by progressive degeneration of upper and lower motor neurons. The main manifestations of upper motor neuron involvement are spasms, muscle stiffness, hyperreflexia, and pathological reflexes, whereas muscle weakness and atrophy are the main signs of lower motor neuron involvement (1). The development of ALS is rapid, and patients often die from respiratory failure within 1–5 years after symptom onset, with a median survival time of 30 months (2). Although some familial cases of ALS can be attributed to single-gene mutations, 90% of the cases are sporadic. However, the etiology and pathogenesis of this disease are complex (3). Immune disorders, inflammation, redox imbalance, autophagy dysfunction, and impaired iron homeostasis are important factors in the progression of ALS (4). A mouse model of ALS revealed that the immune disorders characterized by dynamic changes in inflammatory mediators, such as cytokines and immune cells are elevated before initiation of motor neuron degeneration. In addition, the activation of immune cells and the release of various inflammatory mediators aggravates the loss of neurons and axons, which evidenced the role of inflammatory components in promoting motor neuron death in ALS.

Persistent motor neuron injury in the central nervous system (CNS) is often accompanied by the participation of non-nerve cells, which is characterized by an inflammatory response, such as the activation of microglia, proliferation of astrocytes, infiltration of T lymphocytes and macrophages, and overexpression of inflammatory cytokines (4, 5). A mouse model of ALS with superoxide dismutase 1 (SOD1) revealed that the activation of microglia and the complement system on the motor endplate existed prior to symptom onset, suggesting that inflammation may promote disease progression in ALS (4). Conversely, the studies in patients with ALS and its rodent models have shown that inflammatory cells essentially have dual effects, i.e. anti-inflammatory and inflammatory effects on neurons according to the disease stages (6). This partly explains the ineffectiveness of traditional anti-inflammatory treatments in ALS. Another factor limiting the evaluation of the efficacy of anti-inflammatory therapy is the lack of objective biomarkers of disease activity in human biological fluids (blood, cerebrospinal fluid, urine), especially biomarkers that can track the inflammatory change degree. Molecules from non-nerve cells such as microglia, astrocytes, or macrophages have received increasing attention as potential inflammatory markers.

The purpose of this review is to elucidate the latest progress in applying inflammatory biomarkers in ALS for diagnosis and treatment in the past 10 years, with an emphasis on chitinase, cytokines, acute phase reactive protein, and several rare inflammatory mediators. Furthermore, we summarized the therapeutic strategies *via* inhibiting inflammation and improving immune dysfunction which can help in understanding the potential of these biomarkers and identifying new therapeutic targets to improve the treatment of patients with ALS, prolong their survival, and improve their quality of life.

## Chitinase

Chitinase is a hydrolytic enzyme that is widely presented in nature. It mainly participates in the metabolism of chitin in organisms that contain chitin, such as arthropods, nematodes, bacteria, and fungi (7). Although mammals lack endogenous chitin or chitin synthase genes, they can still express chitinase, which has enzymatic activity, and chitinase-like proteins (CLPs) that are related to homologous structures (8). Based on the similarity of amino acid sequences, human chitinase is classified into the 18-glycosylhydrolase (GH18) family, chitinase (CHIT1), acid mammalian chitinase (AMCcase), and chitinase-like proteins (9). Here, the relationships between chitinase-3-sample 1 (CHI3L1), chitinase-3-sample 2 (CHI3L2), and ALS are discussed mainly.

The major physiological function of chitinase in the human body is to play a role in defense and scavenging by combining chitin and chitin-like polymers. Although chitin enzyme-like proteins have no enzymatic activity, they still bind to chitin with high affinity and participate in a large number of biological processes (10). Given their physiological characteristics of immunomodulation and binding to intracranial titin-like polymers, chitinase and CLP are widely described as markers of neuroinflammation and reactive glial cell activation in various neurological diseases (9, 11). CHIT1, CHI3L1, and CHI3L2 have been used as biomarkers to quantify the response degree of glial cells in clinical trials of drugs that inhibit glial cells’ activity, thereby assessing the relevance of these targets (12, 13). Herein, we review the roles of CHIT1, CHI3L1, and CHI3L2 in ALS to determine their potential value as biomarkers.

### Chitotriosidase (CHIT1)

CHIT1 was the first chitinase found in humans, and its presence has been confirmed in the macrophages of patients with hypermetabolic disease (14). Analysis of cerebrospinal fluid (CSF) samples from patients with various neurodegenerative diseases revealed that the concentration of CHIT1 was higher than that in healthy controls, and the concentration of CHIT1 in patients with ALS was also higher than that in patients with frontotemporal lobe degeneration (FTD), Alzheimer’s disease (AD), and Parkinson’s disease (PD) (15). Therefore, CHIT1 can be used as a potential marker to distinguish patients with ALS from healthy controls and to differentiate ALS from other neurodegenerative diseases. A study by Chen et al. in 2016 confirmed this conjecture, setting the critical value of CHIT1 in CSF at 1593.779 ng/L to distinguish between ALS patients and controls, with a sensitivity of 83.8% and a specificity of 81.1% (16). A recent study of the source of differences in CHIT1 concentration in different neurodegenerative diseases showed that the activation and proliferation of microglia and astrocytes can regulate the concentration of CHIT1 in CSF and further affect the release of proinflammatory cytokines and the loss of motoneurons. It is suggested that the concentration of CHIT1 and the activation of glial cells are the key factors leading to motor neuron degeneration. The observation supported the specific role of CHIT1 in promoting the development of neuroinflammation in ALS (17, 18). A series of studies on CHIT1 corroborated previous findings that inflammatory components are involved in the death of motor neurons, and suggested that CHIT1 expression levels correlate with ALS progression and prognosis, i.e., higher CHIT1 levels lead to shorter survival time. In addition, an interesting study in 2021 found that CHIT1 was significantly negatively correlated with the respiratory function index of forced vital capacity (FVC), which is often used to measure potential respiratory damage in patients with ALS and is a predictor of survival and disease progression (19). Therefore, these studies emphasized the idea that measuring CHIT1 has advantages with regard to monitoring disease progression, predicting survival time, and potentially evaluating treatment response in patients with ALS. However, a study in 2018 reported that, although CHIT1 was significantly correlated with the rate of disease progression (PR), these correlations were not continuous when patients were stratified according to the PR (20). However, Chen et al. demonstrated no significant difference in CHIT1 levels in patients stratified according to PR. Therefore, it is necessary to further explore its potential value in neuroinflammation to identify the triggers of CHIT1 release and cellular expression at different stages and to study its therapeutic efficacy as a key target of neuroinflammatory intervention in the early stages of ALS (16).

### Chitinase 3-like 1(CHI3L1)

CHI3L1 (also known as YKL40) is a 40 kD glycoprotein that was first found in the culture medium of chondrocytes and synovial cells (21). CHI3L1 is associated with inflammation, injury, tissue remodeling, and abnormal cell proliferation, and plays a role in neurodegenerative diseases (22). CHI3L expression is increased in the CSF of patients with ALS, AD, and FTD, but not in those with PD and Lewy body dementia. Therefore, CHI3L1 has received increasing attention as a new biomarker in neurodegenerative diseases (23). Compared to controls, the immune response of patients with ALS is limited to glial fibrillary acidic protein (GFAP)-positive astrocytes in the frontal cortex and spinal cord. However, patients with ALS also exhibit increased expression of CHI3L1 in the anterior horn and motor cortex of the spinal cord, which indicates that the CHI3L1 level in the CSF is related to the symptoms of superior motor neurons. Reactive astrocytes are the main source of CHI3L1 in the CNS. In addition, a comparison study between patients with ALS and FTD in 2018 displayed that the ratio of soluble β fragments of amyloid precursor protein (sAPPβ) and CHI3L1 was directly related to the thickness of the frontotemporal cortex. Furthermore, the Edinburgh cognitive and behavioral ALS screen (ECAS) confirmed that the level of CHI3L1 was associated with cognitive impairment (17). Based on the above results, the level of CHI3L1 in CSF appears to increase with time. Moreover, it is positively correlated with disease progression rate (DPR) and negatively correlated with survival rate, which confirms the value of CHI3L1 in evaluating the prognosis of ALS (24). However, there was no significant difference in CHI3L1 levels reported between asymptomatic mutation carriers and controls, and there was also no significant difference in CHI3L1 levels between pre- and late-stage, and early-stage symptoms (17, 25). Although the release of CHI3L1 confirms the involvement of astrocytes in ALS and the dose-induced neurotoxic effects, the idea that CHI3L1 has an apparent pathogenic function early in the disease remains controversial.

Therefore, additional studies are needed for further clarification of the pathological triggers that induce CHI3L1 release, and for longitudinal evaluation of the correlation between CHI3L1 levels and different clinical parameters at various disease stages.

### Chitinase 3-like 2(CHI3L2)

CHI3L2 (also known as YKL39) is closely related to CHI3L1; however, unlike CHI3L1, CHI3L2 is not a glycoprotein. It was originally isolated from the culture medium of primary human articular chondrocytes (26). As a pseudochitinase lacking chitinase activity, CHI3L2 retains its chitinase-like ligand-binding properties (27). Similar to CHIT1 and CHI3L1, the content of CHI3L2 in the blood is related to the rate of disease progression. However, Kaplan–Meier estimator and Cox proportional hazards modeling revealed that CHI3L2 can also be used as an independent survival predictor as compared to CHIT1 and CHI3L1 (12, 28). In addition, an interesting study conducted in 2017 found that the level of CHI3L2 in patients with ALS who smoke was positively correlated with the rate of disease progression. Further, CHI3L2 was also significantly upregulated with disease progression. Changes in the expression level of chitinases such as CHI3L2 confirmed that tobacco smoking was a risk factor for ALS, and it mediates disease progression in ALS through neuroinflammation (29). These studies highlight the importance of CHI3L2 in evaluating the prognosis of ALS. However, it is important to note that, although CHI3L2 can be detected in macrophages, tumor cells, and even in nerve cells in the cerebral cortex, the expression of CHI3L2 in patients with ALS has not been reported yet. Hence, the source of CHI3L2 in the CSF of patients with ALS is not clear (30). Besides, a longitudinal assessment conducted by Gray et al. in 2020 showed that there was no significant difference in CHI3L2 level between the early and late stages prior to symptom onset as well as no significant fluctuation throughout the entire course of the disease (17). Combined with the above findings, CHI3L2 has some limitations in evaluating the role of neuroinflammation in ALS. Although the interaction between CHI3L2 and neurons and glial cells is not clear, CHI3L2 remains a promising biomarker for the prognosis of ALS. Based on the reports discussed above, it is necessary to further clarify the cell source of CHI3L2 in patients with ALS and the mechanism of action of CHI3L2 in immune response and inflammation.

**Figure 1:**
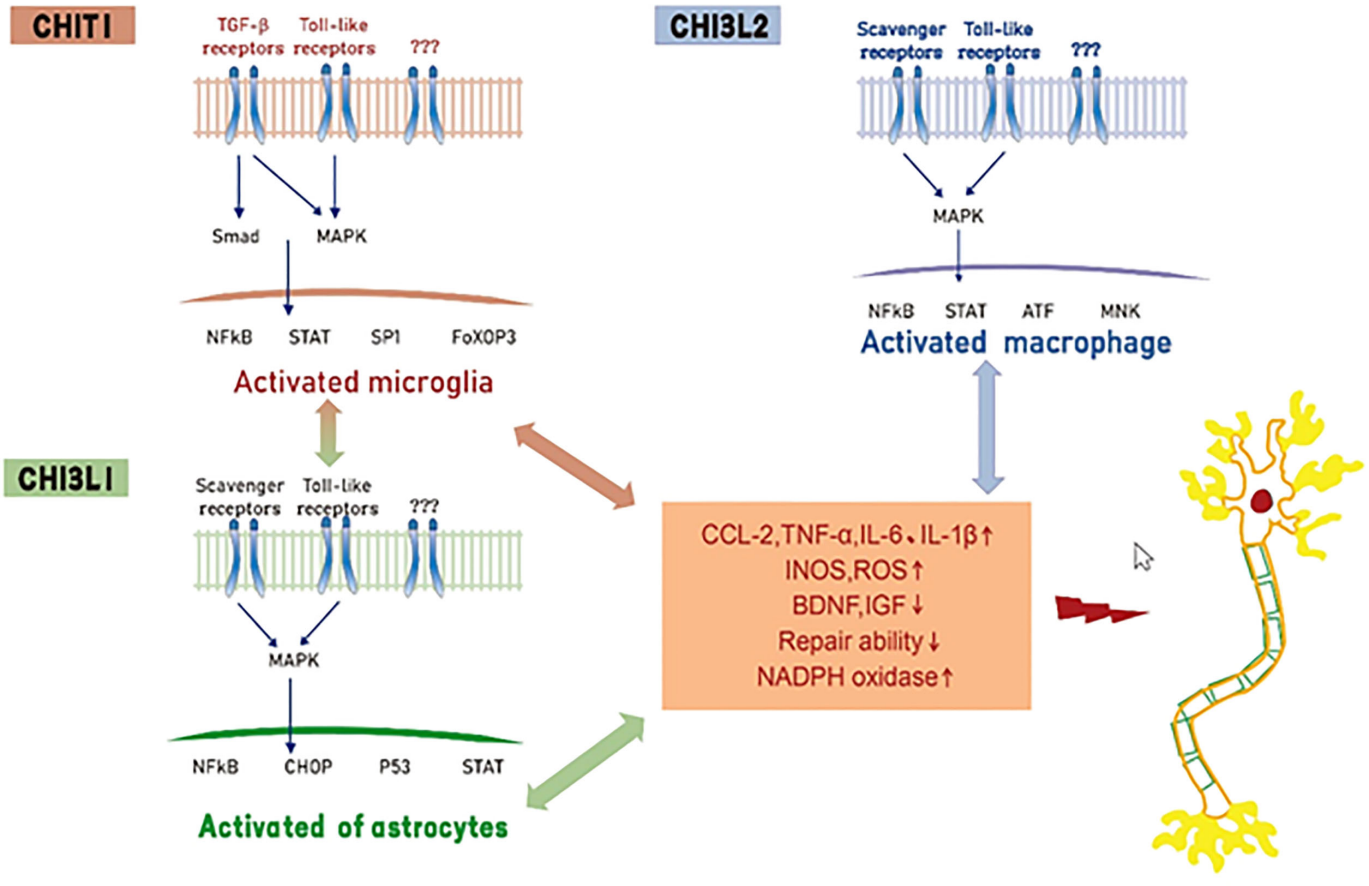
Hypothesis of mechanisms of Chitinase induced neurotoxicity in ALS. CHIT1, CHI3L1 and CHI3L2 activate glial cells and macrophages by interacting with receptors (e. g. toll-like receptors, transforming growth factor beta or scavenger receptors), resulting in the release of pro-inflammatory mediators, inhibition of neurotrophic factor synthesis and decreased tissue repair function.

At present, a consensus reached based on several experimental studies is that CHIT1, CHI3L1, and CHI3L2 produced in the process of neuronal degeneration, have complex functional signal networks that jointly drive the interaction between biological signal molecules in neuroinflammation and the disease microenvironment. However, none of these three biomarkers can capture the whole process of neuroinflammation. It can be assumed that CHIT1 mainly activates microglia and releases pro-inflammatory mediators. At the same level, activated microglia can stimulate astrocytes, resulting in further enhanced microglia and astrocyte responses. CHI3L1 acts on astrocytes and activated astrocytes to release inflammatory mediators, leading to motor neuron degeneration. We hypothesized that CHI3L2 can induce motor neuron degeneration by stimulating macrophages that infiltrate the blood-brain barrier (BBB) and then transform into pro-inflammatory phenotypes (**Figure 1**). Although these three biomarkers cannot replace neurofilament in the diagnosis and evaluation of ALS prognosis, they still have high complementary value for early diagnosis of ALS and differentiation of subtle differences between different neurodegenerative diseases (**Table 1**).

**Table 1:** Association of cerebrospinal fluid chitinase and prognostic evaluation parameters in ALS patients ALSFRS-R, ALS functional rating scale- revised; CHIT1, Chitotriosidase; CHI3L1, Chitinase 3-like 1; CHI3L2, Chitinase 3-like 2; DPR, disease progression rate.

Biomarker	Prognostic evaluation parameters	Positive↑/negative↓association	Reference
**CHIT1**	ALSFRS-R	↓	(18, 20, 31, 32)
	DPR	↑	
	Disease duration	↓	
	Survival	↓	
**CHI3L1**	ALSFRS-R	↓	(11, 24, 31, 33)
	DPR	↑	
	Disease duration	↓	
	Survival	↓	
**CHI3L2**	ALSFRS-R	↓	(12, 29, 31)
	DPR	↑	
	Disease duration		
	Survival	↓	

## Cytokines

Cytokines are small soluble polypeptide proteins secreted by immune cells and histiocytes that regulate cell growth, differentiation, and immune responses by binding to their receptors. There are more than 300 known cytokines, including chemokines, interleukins, interferon (IFN), and tumor necrosis factor (TNF) (34, 35). When they participate in immune and inflammatory responses, such as antigen presentation, cell recruitment, expression of adhesion molecules, and innate immunity, these cytokines play an efficient and synergistic role. The immune network, which is composed of different cytokines, can regulate immune and inflammatory responses in different areas of the same organ. Further, it has different immunomodulatory roles. For example, in neurodegenerative diseases, cytokines released by some resident cells of the CNS in the early course of the disease can counteract inflammatory damage by limiting inflammation or promoting tissue remodeling; however, as the disease progresses, cytokines released by invasive immune cells and some glial cells target the CNS and play a neurotoxic role (36–38). Additionally, with the increasing number of studies on neuroinflammation and peripheral inflammation in ALS in recent years, multiple studies have reported abnormal changes in a variety of cytokines and chemokines in the peripheral blood and CSF of patients with ALS. These observations enhance our understanding of their role in the pathogenesis, early diagnosis, and improvement in the treatment of ALS. Therefore, some researchers suggest that cytokines should be used as biomarkers for the diagnosis and evaluation of the prognosis of ALS. Considering that a large number of cytokines are involved in the disease process, this review cannot be all-inclusive. Instead, we focus on only the key cytokines related to the neuroinflammation in ALS and aim to integrate them into a conceptual framework for comprehensive analysis.

### Interleukins

Interleukin (IL) is a type of cytokine that mediates the interaction between leukocytes and other cells and is synthesized by T cells, macrophages, and endothelial cells etc. Interleukins promote the proliferation, differentiation, and activation of immune and inflammatory cells (39). It has been reported that in patients with ALS, the levels of a large number of interleukins in the blood or CSF were increased compared with a healthy control group or patients with other non-inflammatory neurological disorders (OND), which include IL-1β, IL-2, IL-4, IL-6, IL-8, IL-10, IL-12, IL-13, IL-15, IL-17, IL-18, IL-21 and IL-23 (40–44). Among all cytokines, IL-6 has a high reference value as a biomarker for assessing patient prognosis. Comparing ALS with other neurodegenerative diseases showed that all cytokines, except IL-8, were differentially elevated in AD, PD, or FTD. Among them, only IL-8 was specifically elevated in ALS and not in other neurodegenerative diseases. Moreover, IL-6 has been identified as an astrocyte-dependent biomarker that can evaluate the prognosis of patients with ALS (45). A recent study found that a decrease in the compound muscle action potential (CMAP) amplitude in the phrenic nerve was associated with increased IL-6 levels in ALS (46). In corroboration with previous studies, it also reported that changes in peripheral blood PaO_2_ affected fluctuations in IL-6 levels in the serum and CSF of ALS (47). Therefore, the level of IL-6 reflects the severity of respiratory function involvement in patients with ALS to some extent (46, 47). Simultaneously, Sun et al. also reported that the levels of IL-6 and IL-2 were positively correlated with the severity of muscular dystrophy and negatively correlated with the ALSFRS-R score (48). In patients with ALS with a duration of illness of less than 12 months, IL-6 levels were negatively correlated with disease progression, while in patients with an illness duration of more than 12 months, the IL-6 levels were positively correlated with disease development, which further highlights the prognostic value of IL-6 in assessing ALS (46, 48). Other interleukins that may potentially be implicated in ALS include IL-4, IL-8, IL-13, IL-15 and IL-18, elevated levels correlate with DPR and ALSFRS-R score (42, 49–53). In ALS patients and animal models, levels of IL-2, IL-5, IL-8 and IL-12 were higher than in controls and were associated with shorter survival times and faster disease progression (52, 54–56).

In summary, the involvement of interleukins in the inflammatory processes of ALS has a unique value in evaluating the prognosis of ALS. However, interleukins are often released irregularly in the inflammatory response in ALS, and their relationships with various clinical parameters were contradictory across different studies (48). Therefore, more studies are needed to further clarify the pathological triggers that affect the release of interleukins and the mechanism of crosstalk between interleukins and glial cells.

### Tumor necrosis factors

Tumor necrosis factor (TNF) is classified as TNF-α and TNF-β according to its origin and structure. TNF-α is the mainstay of research on ALS. It participates in cell proliferation and differentiation, phagocytic activation, and cytokine production, and is mainly released by astrocytes and microglia in the CNS, and macrophages in the periphery (57). Some studies have shown that the levels of TNF-α in the CSF and peripheral blood of patients with ALS were higher than that in controls and the levels were positively correlated with the course of the disease (58). It has been reported that the protective or toxic effect of TNF-α on motor neurons depends on activating its two different receptors, whereby activation of TNF receptor (R) 1 can promote the expression of neurotrophins and mediate neuroprotective effects, while activation of TNFR2 can induce neuronal degeneration and play a neurotoxic role (59). Higher levels of TNFR superfamily members (TNFRSF) 1A (CD120), TNFRSF8L (CD30L), TNFRSF18 (GITR), TNFRSF19 (TROY), and TNFSF11 were seen in patients with ALS and the SOD1G93A mouse model compared to controls. However, TNFRSF18 (GITR) levels were lower in the early stage of the disease compared to the control group, and the levels of both TNFRSF18 and TNFRSF19 were negatively correlated with the survival rate (55, 60). Based on these findings, we consider that inhibiting the expression of TNFR2 or administering a modified form of TNF-α may play a potential therapeutic role in patients with ALS during the early stage.

### Chemokines

Chemokines are small proteins that induce directed migration, activation, and development of immune cells. According to their structural characteristics, more than 50 chemokines can be divided into four subfamilies: CXC, CC, C, and CX3C (61). There is growing evidence that some chemokines play key roles in certain stages of ALS as important mediators in inflammatory networks (62, 63). Among all chemokines, CCL2 is considered a sign of non-neuronal cells participating in ALS. In the CNS, the main source of CCL2 may be glial cells, while in the peripheral, T cells, NK cells, and macrophages are sources of CCL2 (64, 65). Importantly, CCL2 activates microglia, which then produce large amounts of pro-inflammatory cytokines and inducible nitric oxide, thereby prompting the recruitment of T cells, NK cells, and macrophages to the CNS (66, 67). Thus, the upregulation of CCL2 can be used as a marker of neuroinflammation and peripheral immune response (68). In addition, the level of CCL2 is related to the destruction of the BBB and positively correlated with the protein level in CSF, which supports the role of CCL2 as a disease-aggravating factor in the neuroinflammation of ALS (67). In addition to CCL2, higher levels of other chemokines have been seen in the blood and CSF of patients with ALS when compared to controls, such as C-C motif chemokine ligand (CCL)3, CCL4, CCL11 (or Eotaxin-1), CCL19, CCL21 (or 6Ckine), C-X-C motif chemokine ligand (CXCL)8, and CXCL10 (43, 50, 60, 67, 69–72). Further analysis of the prognostic value of various chemokines revealed that the levels of CCL2, CCL3, CCL4, CCL11, CXCL8, and CXCL10 in the blood correlated with ALSFRS-R score and DPR. CCL4 and CXCL10 levels positively correlated with ALSFRS-R score and negatively correlated with DPR, and CCL3 and CCL11 levels were negatively correlated with survival time (11, 31, 47, 51, 67). However, other studies have reported that CCL11 can play a neuroprotective role in ALS and was positively correlated with survival time (55). Recent studies report that in contrast to other chemokines, CCL5 level is increased in the CSF of patients with ALS but is lower in blood as compared to the controls (52, 73). Therefore, further grouping studies on the functional characteristics and degree of fluctuations in chemokines are still needed, especially for different specimen types (serum or CSF) and different disease stages, in order to further explore the crosstalk between peripheral immunity and CNS inflammation in ALS.

### Interferons (IFN)

Interferons are classified into type I and type II according to their source, structure, and biological properties, where type I includes IFN-α (with 13 subtypes), IFN-β, IFN-κ, IFN-ϵ, and IFN-ω, while type II interferons are IFN-γ (74). To date, the only interferon that has been found in the body fluids of patients with ALS is IFN-γ, which is produced by microglia, astrocytes, and motoneurons in the CNS as well as by T cells and NK cells in the peripheral NS. Its main function is to activate macrophages and to promote the expression of MHC molecules, antigen presentation, and regulation of cell differentiation. The longitudinal evaluation of IFN-γ in patients with ALS in Northern India by Babu et al. revealed that the levels of IFN-γ in the CSF and blood were higher than that in the control group, and IFN-γ increased gradually with the progression of the disease, reaching a peak at 24 months after disease onset (75). A study of patients with ALS by Liu et al. further demonstrated that the level of IFN-γ in CSF was consistent with disease progression throughout the course of ALS, whereas the level of IFN-γ in the serum was only related to disease progression in the early stage of the disease (76). It has been suggested that IFN-γ in the CSF is a more reliable biomarker for diagnosing and monitoring disease progression than that IFN-γ in serum. Although many studies have revealed the diagnostic value of IFN-γ in ALS, the levels of IFN-γ in the CSF and blood of part patients with ALS are lower than those in controls. Hence, the role of IFN-γ as a potential biomarker in ALS remains controversial (40, 77).

### Colony-stimulating factors (CSFs)

Colony-stimulating factors (CSFs) are cytokines that stimulate the proliferation and differentiation of pluripotent hematopoietic stem cells and hematopoietic progenitor cells at various stages of differentiation. Here we focus on the role of G-CSF and GM-CSF in ALS. G-CSF and GM-CSF levels in the CSF and blood of patients with ALS are higher than that in controls (70, 78). In addition, the concentration of plasma GM-CSF decreased, accompanied by a high level of GM-CSF in the CSF when the disease progressed, confirming that the concentration of plasma GM-CSF negatively correlates with the duration of the disease (71). This may be due to a change in BBB permeability, which would result in an increase in plasma GM-CSF transported to the CSF. GM-CSF can also act on neurons by upregulating the apoptotic molecules (B-cell lymphoma 2) Bcl2 and (B-cell lymphoma xL) BclXL; hence, an increase in GM-CSF level in the CSF may be related to neuroprotection. In summary, GM-CSF has potential as a prognostic marker for ALS (71, 79).

### Other cytokines in ALS

Vascular endothelial growth factor (VEGF) is a neurotrophic cytokine that is induced by hypoxia (80). VEGF was elevated in the CSF and blood of patients with ALS, especially in patients with symptom onset in the limbs and a longer course of disease before the first hospitalization, which is considered to be related to the slow progression of the disease (31). In patients with rapid disease progression and a short survival period, VEGF levels were significantly lower than those in the control group, and VEGF levels were positively correlated with the ALSFRS-R score and disease duration (70, 81). Hypoxia can induce the overexpression of VEGF in the CSF and VEGF plays a neuroprotective role in preventing neuronal apoptosis (31). A study conducted by Moreau et al. found that VEGF in the CSF of ALS patients had a paradoxical response to hypoxia; ALS patients with hypoxemia have lower upregulated levels of VEGF than controls who also had hypoxemia. Moreover, the severity of hypoxemia in ALS patients is negatively correlated with VEGF levels, while the opposite was true in controls, suggesting that impaired regulation of hypoxia in ALS patients is closely related to VEGF (82). The above observations support the use of VEGF as an alternative therapeutic agent and neuroprotective factor to ameliorate the adverse effects of hypoxia in ALS patients, thereby delaying motor neuron degeneration.

Similar to VEGF, as a common neurotrophic factor, the levels of basic fibroblast growth factor (BFGF) and platelet-derived factor (PDGF)-BB in CSF and blood of patients with ALS were significantly higher than those of the control group, and BFGF was positively correlated with survival time and negatively correlated with disease progression rate (52, 70).

Another cytokine involved in the course of ALS is transforming growth factor-β (TGF-β), that can play a role in a variety of biological processes, including angiogenesis, fibrosis, and wound healing (83). It has been found that in SOD1G93A mice, the up-regulation of TGF-β expression preceded the appearance of corresponding symptoms, and the content of TGF- β increased with the progression of the disease (84). When combined with other findings, the levels of TGF- β in plasma and CSF in patients with ALS were higher than those in the control group, and the expression in skeletal muscle was related to the degree of muscle weakness and disease progression (52, 85). However, when glial cells express excessive TGF- β, the neuroprotective effect is shifting into neurotoxicity, thus accelerating neuronal apoptosis (86). Therefore, blocking the signal transduction pathway of TGF- β in a specific way may be a promising target for the development of new therapy for ALS.

Other cytokines were found to be significantly upregulated in a mouse model of ALS, and, unlike the above cytokines already discussed, the upregulation of the expression of these cytokines occurred at the asymptomatic stage, including that of activin receptor-like kinase 1 (ALK-1), cluster of differentiation 30 ligand (CD30L), galectin-1, galectin-3, and VEGFD (87–90). Increased expression of ALK-1 and galectin-1 in the symptomatic stage was negatively correlated with survival time but positively correlated with the rate of progression of the disease (55). Galectin-1 is a member of the galactoside-binding lectin (GBL) family. The content of galectin-1 in the CSF and blood of a mouse model of ALS was higher than that in control mice, especially in mice with rapid disease progression and short survival time, which is negatively correlated with the survival rate of these mice (91). However, based on the clear prognostic value of Galectin-1, it has been reported that Galectin-1 can prevent inflammation-induced neuronal degeneration by inactivating microglia through activation and thus exerting neuroprotective effects (40, 92). The reason for this contradiction may be that the activation of some unknown factors triggers a potential signal transduction pathway and hinders galectin-1’s neuroprotective effect. Therefore, actively studying the expression of galectin-1 in plasma and target tissues and clarifying its pathological triggering factors and the underlying signal transduction pathway are useful methods to further explore the potential value of galectin-1.

**Figure 2:**
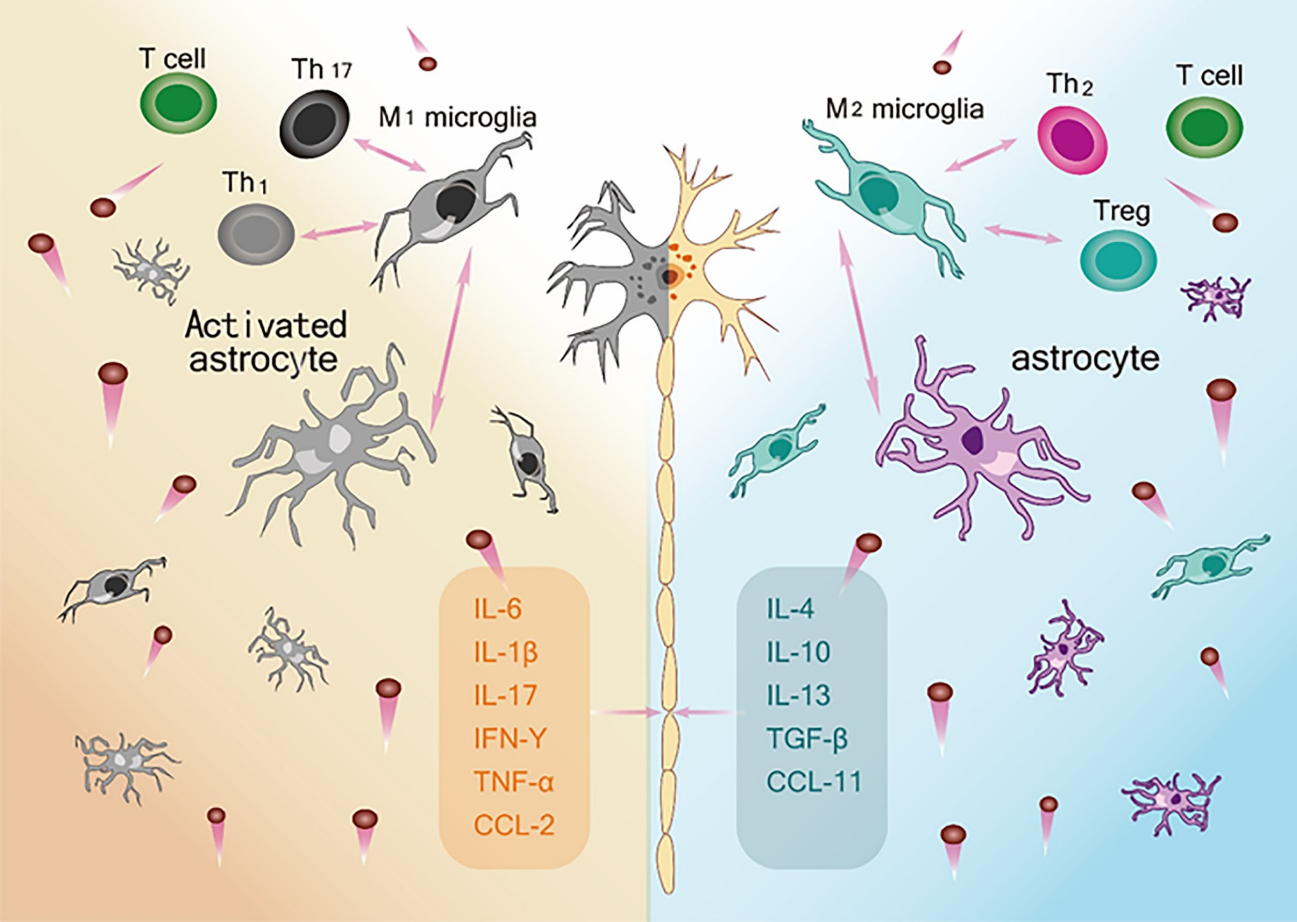
The ambivalent role of Inflammatory network on motor neuron survival. Left: M1 microglia, Activated astrocytes and Th1and Th 17 and their respective secreted pro-inflammatory cytokines constitute an immune network (IL-6, IL-1β, IL-17, IFN-γ, TNF-α, CCL-2) that leads to neurotoxic effects and death of motor neurons. Right: M2 microglia, astrocytes and Th2 and Treg and their respective release of anti-inflammatory and neurotrophic factors (IL-4, IL-10, IL-13, TNF-β, CCL-11) constitute an immune network that supports the function and activity of neurons.

Taken together, the extant literature to date (**Table 2**) supports the view that within the neuroinflammatory network constructed by various glial cells and peripheral immune cells, the neuroprotective and neurotoxic cytokines act as the key nodes of the inflammatory network to regulate disease progression through their interactions (**Figure 2**). In addition, by evaluating the correlations between various cytokine levels and the ALSFRS-R score, DPR, and survival time at various stages of the disease, these studies emphasize the potential value of cytokines as CSF or blood biomarkers in early diagnosis, evaluation of prognosis, and treatment targets. However, the results of all cytokine studies were not completely consistent and were heterogenous. To solve this, it is necessary to determine a combination of biomarkers to improve the understanding of the effects of changes in cytokine activity on neurodegeneration in ALS.

**Table 2:** Association of cerebrospinal fluid Cytokines and prognostic evaluation parameters in ALS patients ALSFRS-R, ALS functional rating scale- revised; CCL, C-C motif chemokine ligand; DPR, disease progression rate; G-CSF, Granulocyte colony stimulating factor, GM-CSF, Granulocyte-macrophage colony stimulating factor; IL=Interleukin; IFN-γ=Interferon gamma; TNF=Tumor necrosis factor; VEGF=Vascular endothelial growth factor.

Biomarker	Prognostic evaluation parameters	Positive ↑/negative ↓association	Reference
**IL-6**	ALSFRS-R	**↓**	(46–48)
	DPR	↓ (Less than 12 months) ↑ (more than 12 months)	
	Disease duration	**↓**	
	Survival	**↓**	
**TNF-α**	ALSFRS-R	**↓**	(59, 93, 94)
	DPR	↑	
	Disease duration	**↓**	
	Survival	**↓**	
**GM-CSF**	ALSFRS-R		(71, 95)
	DPR		
	Disease duration	**↓**	
	Survival		
**IFN-γ**	ALSFRS-R	**↓**	(75, 76, 96, 97)
	DPR	↑	
	Disease duration	**↓**	
	Survival	**↓**	
**CCL2**	ALSFRS-R	**↓**	(11, 31, 47, 51, 67)
	DPR	↑	
	Disease duration	**↓**	
	Survival	**↓**	
**VEGF**	ALSFRS-R	↑	(70, 80, 81)
	DPR	**↓**	
	Disease duration	↑	
	Survival	↑	
**G-CSF**	ALSFRS-R	↑	(98, 99)
	DPR	**↓**	
	Disease duration	↑	
	Survival	↑	

## Acute phase protein(APP)

Acute phase protein (APP) is a specific blood protein produced by changes in the metabolic pattern of the liver to maintain the stability of the internal environment and cope with local or systemic disorders caused by trauma or inflammation (100). So far many studies have observed that ALS pathogenesis is often accompanied by fluctuations in APP levels. Therefore, there may be a common mechanism between CNS inflammation, peripheral immune system changes, and APP level fluctuations in patients with ALS. This review discusses soluble CD14 (sCD14), lipopolysaccharide-binding protein (LBP), and C-reactive protein (CRP), focusing on the fluctuating levels of these APPs, and their value in diagnosis and prognosis. Together with other inflammatory markers, they constitute an inflammatory profile to improve the specificity and sensitivity of ALS diagnosis and the value of assessing APP levels in aiding disease diagnosis and prognosis. APP can be combined with other inflammatory markers to form a map of inflammation and improve the diagnostic specificity and sensitivity of ALS.

### Classic acute phase protein

C-reactive protein (CRP) is a pentamer composed of five identical subunits that bind to phosphocholine (PCH) in a calcium-dependent manner. As an APP, CRP is an important component of the acute phase response. It can be produced in the liver and then transported to other organs through the circulatory system. Furthermore, it has also been proven by molecular genetics techniques that CRP can be produced by neurons in the brain. In particular, upregulation of CRP in areas of the brain damaged by neurodegenerative diseases was most evident (101). CRP is not only a sensitive marker of systemic inflammation but also an activator of microglia. Upregulation of CRP expression can alter the permeability of the BBB and induce microglial activation. With the gradual increase in the inflammatory response during the progression of ALS, activated microglia can further promote the degeneration of motor neurons (102, 103). Therefore, in view of the potential inflammatory processes involved in the pathogenesis of ALS, researchers have proposed the use of CRP as a possible disease biomarker for the early diagnosis and evaluation of prognosis of ALS. A study in 2011 found a significant difference in the ratio of phosphorylated neurofilament heavy chains to CRP in patients with ALS as compared with patients with other neurodegenerative diseases and healthy controls, indicating that CRP has high specificity as a diagnostic biomarker (104). Further, to investigate diagnostic accuracy and sensitivity with CRP, Ryberg et al. conducted a study to evaluate the accuracy of measuring CRP for the diagnosis of ALS with 9 mg/ml as the cut-off value and observed that the total accuracy of distinguishing patients with ALS from healthy controls according to the level of CRP in the CSF was 62% (105). Meanwhile, Kharel et al. conducted a combined frequency analysis of several studies and reported that approximately 53% of ALS patients had statistically significant increases in CRP levels compared to healthy controls (106). In summary, these studies confirmed the superiority of measuring CRP levels in the diagnosis of ALS. However, with the increasing number of studies on CRP and neuroinflammation, the link between CRP and ALS has been questioned owing to studies with negative results; therefore, extensive cohort studies are still needed to evaluate its value in the early diagnosis of ALS.

Considering the value of CRP in evaluating disease prognosis, a multicenter cohort study of patients with ALS in Italy found that CRP levels in patients were lower before symptom onset, but gradually increased with the progression of the disease, especially in the months before death, indicating that CRP levels were positively correlated with disease progression (107). Subsequently, 50 patients were randomly selected and followed up for 1 year. CSF CRP level was positively correlated with the severity of neurological functional impairment and negatively correlated with the ALSFRS-R score. Accordingly, Sun et al. evaluated the correlation between CRP levels and survival time, and observed that patients with CRP levels higher than the median had a higher mortality rate; that is, higher CRP levels in the CSF were associated with a shorter survival time (108). In addition, CRP levels can be used as a valuable index to evaluate the fluctuation of the disease and to predict possible respiratory tract infections during the course of the disease (109). These studies confirmed the value of CRP as a prognostic biomarker in patients with ALS. As a relatively easy-to-obtain biomarker of prognosis, CRP has certain advantages, especially for patients with ALS with rapid disease progression, given the lack of effective evaluation methods. CRP can also be used as a biomarker to evaluate the therapeutic effects of drugs in clinical trials. In 2017, Lunetta et al. used serum CRP levels as a stratified biomarker to further analyze the results in the phase II trial of NP001 (107). Patients with elevated CRP levels significantly slowed the progression of neurological functional impairment after receiving high-dose NP001 treatment (110). In addition, by measuring the changes in the ALSFRS-R score, it was found that compared with the placebo group, the functional deterioration in patients with elevated CRP levels decreased, and these changes showed significant NP001 dose-dependent characteristics. The use of CRP as a sensitive inflammatory marker to stratify patients with ALS will be helpful to fully exploit the potential value of anti-inflammatory therapies.

### Other acute phase proteins

In addition to the classical APP-CRP, the serum levels of other APPs, such as soluble CD14 (SCD14) and LBP, in patients with ALS were also significantly higher than that in controls (111). However, unlike CRP, which classifies ALS patients into fast- and slow-progressive types, SCD14 and LBP are only elevated in the serum of patients with fast- or rapid-progressive disease, suggesting that SCD14 and LBP levels are associated with a faster rate of disease progression and a shorter survival time (112). A positive correlation between LBP and SCD14 has been reported; that is, LBP is increased with an increase in SCD14 level (112).

As the biomarker of ALS, APPs have a potential value for diagnosis and prognosis, and can also be used to stratify patients (**Table 3**). Most studies on APPs used blood samples, which were obtained easily. APP, as a non-specific marker of systemic inflammation, has 62% diagnostic accuracy and shows good sensitivity in patients with ALS, but most samples were taken from CSF (114). Because serum APP levels are susceptible to interference by the patient’s underlying disease, cardiovascular risk factors, recent infections, trauma, other inflammatory diseases of the peripheral nervous system, and the application of inflammation-sensitive drugs, we remain uncertain about the diagnostic value of APP when CSF is observed along with serum samples. In addition, given the diversity of changes in APP levels in body fluids, analysis of larger cohorts of ALS patients, expansion of sample types, and longitudinal evaluation of samples within them are needed to clarify how APP responds to disease status and how APP changes across disease stages.

**Table 3:** Association of Acute phase protein and prognostic evaluation parameters in ALS patients.

Biomarker	Prognostic evaluation parameters	Positive ↑/negative ↓association	reference
CRP (CSF)	ALSFRS-R	↓	(107, 108, 110, 113)
	DPR	↑	
	Disease duration	↓	
	Survival	↓	
LBP (Serum)	ALSFRS-R	↓	(112)
	DPR	↑	
	Disease duration	↓	
	Survival	↓	
SCD14 (Serum)	ALSFRS-R	↓	(112)
	DPR	↑	
	Disease duration	↓	
	Survival	↓	

ALSFRS-R, ALS functional rating scale- revised; CRP, C reactive protein; DPR, disease progression rate; LBP, Lipopolysaccharide binding protein; SCD-14, soluble CD14.

## Other inflammatory biomarkers

Other inflammatory factors differ between patients with ALS and controls and at various stages of the disease, indicating that these inflammatory factors may be related to the pathogenesis of ALS. The plasma level of clubcellprotein16 (CC-16) was significantly higher in patients with ALS than that in healthy controls and its content was positively correlated with DPR in patients with ALS (110). Furthermore, CC-16, a lung-derived protein, is often used as a biomarker to describe pulmonary dysfunction. For patients with ALS with elevated CC-16 levels, the probability of requiring non-invasive mechanical ventilation within 6 months was greatly increased, along with an increased risk of death (47). It has been suggested that the level of CC-16 has a unique value in evaluating the prognosis of patients with ALS. In contrast to biomarkers of respiratory failure in patients with ALS, CC-16 has no effect on respiratory muscle strength, reflecting the process of pulmonary interstitial inflammation caused by aspiration or poor ventilation in parts of the lung (115). Therefore, CC-16 can be used as an early warning index for respiratory failure in patients with ALS.

Another unpredictable protein during the course in ALS patients and mouse models is immunoglobulin G. Through a series of studies, IgG has shown diagnostic and prognostic value related to ALS (52). The level of IgG glycosylation structure in CSF of patients with ALS was significantly increased, and the predicted value after ROC analysis was similar to that of phosphorylated neurofilament heavy chain (116). Of note, under the background of ALS, there is a unique polysaccharide structure in the Fc region of IgG, whose expression frequency and content in the Fc domain were closely related to clinical progress (116).

Other molecules that change in ALS are CD-5L and Ficolin-3. The level of CD-5L in patients with ALS was higher than that in controls, and the level was positively correlated with survival time, but there is a lack of large-scale cohort studies and longitudinal evaluations to verify this phenomenon (47, 117). However, Mohanty et al. demonstrated no significant correlation between Ficolin-3 levels and various clinical features (muscle stiffness, hyperreflexia, muscle weakness, and atrophy). Therefore, both CD-5L and Ficolin-3 require further study as potential biomarkers of ALS (118).

Interestingly, changes in immune cell expression also have potential value in evaluating the prognosis of ALS. Jin et al. reported that the levels of Th1, Th17, NK cells, and monocytes in patients with ALS increased with the progression of the disease, and the levels of Th1 and Th17 were negatively correlated with the survival time of patients (53). In addition, the studies by Keizman et al. suggest that the neutrophil/lymphocyte ratio (NLR) continued to increase in patients with ALS and is related to the rate of disease progression and the ALSFRS-R score (113).

Longitudinal assessment of changes in microglia and monocytes of spinal cord origin in a mouse model of ALS revealed that the levels of microRNA-124 (miR-124), miR-155, miR-125b, miR-146a, and miR-21 were upregulated in symptomatic mice compared to pre-symptomatic mice, with miR-155 being the most significantly upregulated miRNA. miR-155 was upregulated at an earlier stage of symptom onset compared to other miRNAs (47, 119, 120). MiR-155 is prominent in research on ALS biomarkers. In microglia from SOD1G93A mice, miR-155 together with miR-125b could promote the transformation of microglia to the M1 phenotype, directing the inflammatory response to a pro-inflammatory direction, and thus aggravating neurotoxicity (121). Therefore, inhibiting the activation of miR-155 in mouse models of ALS can significantly prolong the survival of mice. Further, the study found also that miR-155 had the highest expression level in spinal cord tissues of patients with Sporadic Amyotrophic lateral sclerosis (SALS) and Familial Amyotrophic lateral sclerosis (FALS). Therefore, an increasing number of studies have focused on miR-155 as a therapeutic target (122).

The intermediate products of the kynurenine pathway (KP) are involved in the neuroinflammatory process of ALS, which provides a unique value for the early diagnosis and prognosis of ALS as well as a new and potentially effective target for the treatment of ALS (123, 124). One study focused on quinolinic acid (QUIN), tryptophan (TRP), picolinic acid (PIC), and kynurenine (KYN), all of which had higher levels in the CSF of patients with ALS as compared to controls (125). Interestingly, another study showed that QUIN and TRP, which are neurotoxic, were positively correlated with the severity of ALS symptoms, while PIC, which is a neuroprotective agent, was positively correlated with the survival time in a mouse model of ALS (126). IIzecka et al. demonstrated that the level of Kynurenic acid (KYNA) was lower in the early stage of the disease and then gradually increased with disease progression, with significantly higher levels in the CSF of ALS patients with medullary onset compared to ALS patients with limb onset (127).

## Inflammatory biomarkers in ALS: Challenges and future

The irreplaceable value of inflammatory molecules or cell selection as biomarkers in the pathogenesis and prognosis of ALS is relatively clear (**Tables 1**–**4**). The evidence provided by inflammatory biomarkers in human and experimental models largely replicates the prediction of disease development by human neuroimaging, neuro-electrophysiology, and pathological biopsies, which suggests that neuroinflammation caused by non-nerve cells can aggravate neuronal dysfunction, and different inflammatory molecules can form an immune network to affect disease progression by regulating the balance between anti-inflammatory and pro-inflammatory. However, it is impossible to solely rely on single inflammation-related molecules or proteins to diagnose, predict disease progression, and evaluate the effectiveness of clinical trials.

**Table 4:** Association of other inflammatory factors and prognostic evaluation parameters in ALS patients ALSFRS-R, ALS functional rating scale- revised; CD-5L, cluster differentiation 5 ligand; CC-16, club cell protein 16; DPR, disease progression rate; QUIN, quinolinic acid, TRP, tryptophan, PIC, picolinic acid.

Prognostic evaluation parameters	Association	Biomarker	Reference
ALSFRS-R	Positive ↑	CD-5L	(117)
	Negative ↓	CC-16; miR-155	(110, 120, 121)
DPR	Positive ↑	CC-16; IgG; miR-155; QUIN; TRP	(110, 121, 125, 128)
	Negative ↓	PIC	(129)
Disease duration	Positive ↑	CD-5L	(117)
	Negative ↓	miR-155	(121, 130)
Survival	Positive ↑	CD-5L; PIC	(117, 129)
	Negative ↓	CC-16; Th1; Th17; miR-155	(53, 110, 113, 130)

In order to solve this problem, some researchers proposed that it is more appropriate to choose a combination of several biomarkers than focusing on a single one. For instance, the combination of gene expression from the same cell with different inflammatory factors to jointly evaluate ALS, which is conducive to targeting some inflammatory pathways, even tracking disease processes further upstream. Besides, this combined assessment with certain clinical parameters (ALS type, anatomical location of motor neuron damage, sex, age, etc.) can identify inflammatory features and subgroups that are more sensitive to selected treatment, for example, upper motor neuron damage. But the inflammatory pattern of sporadic ALS may be significantly different from that of patients with familial ALS.

As the genetics of inflammation expands and the large international database on ALS deepens, a specific direction of development is to apply a combination of biomarkers from different pathways to enhance the insights gained from a limited number of ALS patients, leading to multivariate analysis and longitudinal assessment of clinical trajectories for several candidate biomarkers of clear value. Meanwhile, the correlation between the combination of candidate biomarkers and some clinical parameters needs to be determined. Finally, improving the clinical conversion efficiency of biomarkers, promoting future multi-drug trials, and developing personalized and accurate drug therapy are the next three steps for clinical utility.

## Targeting inflammatory biomarkers as therapeutic approaches

### Targeting cytokines

As the main cytokine of the inflammatory response, IL-6 plays an important role in the regulation of metabolic disorders and neuroinflammation; therefore, it is considered a therapeutic target for ALS (45). A recent preclinical study showed that knockout of the IL-6 gene or blocking the IL-6 pathway has obvious anti-inflammatory effects, increasing the number of regulatory T cells in the blood and a decrease in the concentration of the pro-inflammatory chemokine CXCL-1. Unfortunately, it could not significantly improve the loss of motor function in mice, but rather accelerated the weight loss of the mice (131). The reason may be that blocking the IL-6 pathway has an impact on many parameters, especially leading to metabolic disorders that change body weight and disease progression. In addition to IL-6 and IL-33 can also exert anti-inflammatory effects by regulating peripheral T cells, inhibiting disease progression (132). However, in a mouse experiment with IL-33, intraperitoneal injection of IL-33 into female mice could significantly delay disease progression, while male mice did not respond to treatment (133). This suggests that there may be other potential mechanisms that cause the anti-inflammatory effects of IL-33 to be sex-dependent.

Unlike IL-33, which mediates its anti-inflammatory effects by regulating the peripheral immune system, G-CSF can not only modulate anti-inflammatory polarization by regulating inflammatory cells and other cytokines, but also mobilize hematopoietic stem cells to interact with local cells to produce neurotrophic factors, thus playing a neuroprotective role (98). The routine administration of G-CSF involves repeated daily injections of filgrastim. A clinical trial of G-CSF in 36 ALS patients conducted in 2018 found that long-term subcutaneous injections of G-CSF were safe for ALS patients and helped improve motor neuron survival, but the correspondence between dose and effect is unclear because of the small sample size (98, 134). Additionally, although G-CSF is safe, long-term use carries the risk of splenomegaly and splenic rupture (135). In contrast to the anti-inflammatory effect of G-CSF, IFN-γ is a strong pro-inflammatory cytokine that can induce neuroinflammation and lead to the death of motoneurons. Therefore, inhibition of IFN-γ activity can ease the inflammatory process and slow disease progression. In a study of ALS mice, micro-pumping of anti-IFN-γ antibodies into the CSF could effectively save motoneurons from IFN-γ-induced death and significantly delay the progression of motor dysfunction in mice (96). Finally, a mathematical model of the cell-cytokine communication network in ALS also predicted that neutralizing IFN-γ activity is an effective therapeutic target (136).

Unlike the neurotoxicity caused by other pro-inflammatory cytokines, the neurotoxicity or neuroprotective role of TNF-α is receptor-dependent (59). Thalidomide and lenalidomide have been used to inactivate TNF-α in SOD1G93A mice, which prolonged the survival time of mice and enhanced exercise ability (137). However, when the results obtained in the mouse model were transformed into patients with ALS, it was found that the patients treated with thalidomide did not receive any beneficial effect, and with an increase in application time and dose, the negative effect gradually appeared (59).

Compared with other cytokines that have a relatively clear mechanism of action, the therapeutic mechanism of ALS targeting galectin-1 is still relatively vague, and the therapeutic effect is more contradictory. Kato et al. found that the number of residual motor neurons in the spinal cord of ALS mice treated with galectin-1 was better than that in untreated mice and that galectin-1 treatment improved the motor ability of model mice, delayed the appearance of symptoms, and prolonged survival time (92). However, with the expansion of the study sample, it was found that there was a positive correlation between the level of galectin-1 and disease progression (91). Therefore, it is necessary to further study the mechanism of galectin-1 to clarify its potential value as a therapeutic target.

Unlike therapies targeting most cytokines, which salvage motor neurons by regulating the balance between pro- and anti-inflammation, therapies targeting VEGF salvage motor neurons by nourishing the nerves and thus delaying the degeneration of motor neurons (80). Research on the therapeutic value of VEGF in ALS has primarily focused on VEGF-A. A mouse model treated with VEGF-A gene therapy and VEGF-A protein therapy showed positive therapeutic effects, which were mainly characterized by prolonged survival time and improved motor function in model mice (138). There is evidence of the potential to develop VEGF-A-based therapies in the future (131).

### Anti-inflammatory therapy by NP001

Unlike cytokines that regulate inflammation *in vivo*, NP001 is a PH-dependent IV formulation of purified sodium chlorite that exerts inflammatory modulating effects *in vivo* and *in vitro* simultaneously (139). NP001 shifts monocytes and macrophages from a pro-inflammatory state to a devouring state by down-regulating the expression of nuclear factor κ B and inhibiting the production of pro-inflammatory cytokine IL-1b. Despite there will be dizziness and pain in infusion sites during ALS treatment, it is secure and well tolerated (110, 140). A six-month preliminary evaluation of the efficacy of NP001 indicated there is no statistically significant benefit to the progression of ALS (107). However, in patients with significant systemic inflammatory response, there is a slow drop in the progression rate of ALSFRS-R in the NP001-treated group, which showed an obvious NP001 dose-dependent profile, compared to the placebo group (110). Future continuing clinical studies for ALS patients with obvious systemic inflammatory responses will fully characterize the potential disease modification effect of NP001.

### Targeting the innate immune system

In addition to treatments targeting cytokines for anti-inflammatory treatment in ALS, the study of miR-155 provides a new direction for treatments targeting the innate immune system (122). Conventional treatment with miR-155 mainly includes intraventricular or peripheral anti-miR-155 therapy and gene ablation miR-155 therapy, in which gene ablation miR-155 can reverse the pro-inflammatory signaling from abnormally activated microglia and monocytes, and inhibit 72% of abnormal protein expression in the spinal cord of SOD1G93A mice, thus delaying disease progression and prolonging the survival time of mice (122, 141). Additionally, the KP pathway is being widely considered as a potential pathogenic factor, wherein the treatment of neuroactive intermediates (QUIN, TRP, PIC, KYN) produced in this process has shown very promising results in clinical trials of ALS (125, 142).

### Targeting non-neuronal cells

The treatment of Treg in ALS patients and mouse models slows disease progression (143). With the extension of the survival time of the model mice, the amplification of Treg levels infiltrated into the CNS which leads to the inhibition of inflammatory activity of astrocytes and microglia. The infiltration turned into a neuroprotective immune response. The gene expression of neurotrophin in the spinal cord and peripheral nerve increased significantly, increasing the number of motor neurons retained in the spinal cord (90). Further, safety and tolerance of Tregs infusion are two other advantages for all patients, hence, this method is becoming an effective strategy in anti-inflammatory therapy for ALS. By releasing TGF and up-regulating Treg and T helper (Th) 2 cells, experimental therapy reported that mesenchymal stem cells (MSCs) promoted the inflammatory balance of ALS patients from pro-inflammatory toxicity to anti-inflammatory and neuroprotective state (144).

## Conclusions and future directions

ALS is not only a pure neuronal degeneration disorder but is also involved in glial cells that maintain neuronal stability and peripheral immune cells that infiltrate the central nervous system. In ALS, attention must be paid to inflammation in both the CNS and peripheral NS. More specifically, attention should be paid to the immune network composed of glial cells and their derived molecules in the CNS, and inflammatory cells and their derived molecules in the peripheral immune system. This paper summarized the neuroinflammatory characteristics of ALS, the expression of related inflammatory genes, the levels of inflammatory molecules such as cytokines or APPs, and the pathophysiological changes of different elements in various stages of the disease. This may guide future research and the therapeutic targeting of inflammatory markers in a new direction (**Table 5**).

**Table 5:** Anti-inflammatory therapeutic strategies of ALS G-CSF, Granulocyte colony stimulating factor; IL, Interleukin; IFN-γ, Interferon gamma;TNF, Tumor necrosis factor; Tregs, Regulatory T cells; VEGF, Vascular endothelial growth factor; QUIN=quinolinic acid, TRP, tryptophan, PIC, picolinic acid; KYN= kynurenine; MSCs, mesenchymal stem cells.

Therapeutic approaches		Description	References
Targeting cytokines	IL-6	Regulating the number of Tregs in the blood and reduces the level of pro-inflammatory cytokine CXCL-1.	(52, 145)
	IL-33	Regulation of peripheral T-cell delivery of anti-inflammatory effects, but sex-dependent anti-inflammatory effects.	(86, 87)
	G-CSF	Regulation of inflammatory mediators and mobilization of hematopoietic stem cells to produce neurotrophic factors	(55, 88, 146)
	IFN-γ	Inhibit the process of neuroinflammation and delay the process of motor dysfunction in mice	(89, 91)
	TNF-α	The survival time of mice was prolonged and the motor ability was enhanced, but there was no beneficial effect on ALS patients.	(40, 51, 147)
	Galectin-1	The treatment mechanism is vague and the treatment effect is contradictory.	(11, 67)
	VEGF	Delay the degeneration of neurons, prolong the survival time of mice and improve motor function.	(52, 69, 101)
Anti-inflammatory therapy For NP001	NP001	It has anti-inflammatory properties and the efficacy is dose-dependent, but it is not statistically beneficial to the progress of ALS.	(78, 148)
Targeting the innate immune system	miR-155	Inhibit the pro-inflammatory signal and abnormal protein expression of inflammatory cells, and then prolong the survival time of mice.	(55, 104)
	KP (QUIN、TRP、PIC、KYN)	Improve the neuroinflammatory process of ALS and open a new door for anti-inflammatory therapy of ALS.	(83, 105)
Targeting non-neuronal cells	Tregs	Inhibit the pro-inflammatory activity of cells and increase the expression of neurotrophic factors, so as to slow down the progression of the disease.	(106, 108)
	MSCs	Activate anti-inflammatory activity and neuroprotective state to inject vitality into anti-inflammatory therapy of ALS.	(102)

Identifying inflammatory pathways and targets related to the progression of ALS, especially in patients selected according to Chit1 or CRP levels, and potential anti-inflammatory therapy may be of immense value. As ALS is a multi-factorial disease, it is still particularly important to actively promote multi-drug and multi-pathway combined therapy, while individualized and accurate drug therapy for key immune factors should be conducted in the future.

## Data availability statement

The original contributions presented in the study are included in the article/supplementary material. Further inquiries can be directed to the corresponding author.

## Author contributions

ZJ is responsible for the preliminary review of the literature, the collation of information and writing. ZW study design, analysis and interpretation of data and revision of the manuscript for intellectual content. XW interpretation of data and revision of manuscript for intellectual content. X-FY directed and supervised the study. All authors contributed to the article and approved the submitted version.
